# Supplementary material for: Multi-omics analysis reveals contextual tumor suppressive and oncogenic gene modules within the acute hypoxic response
Source: Nat Commun. 2021 Mar 2;12:1375. doi: 10.1038/s41467-021-21687-2 (PMC7925689; doi:10.1038/s41467-021-21687-2)
Supplement: Supplementary file 1 — Supplementary Information [file 41467_2021_21687_MOESM1_ESM.pdf]

SUPPLEMENTARY FIGURES

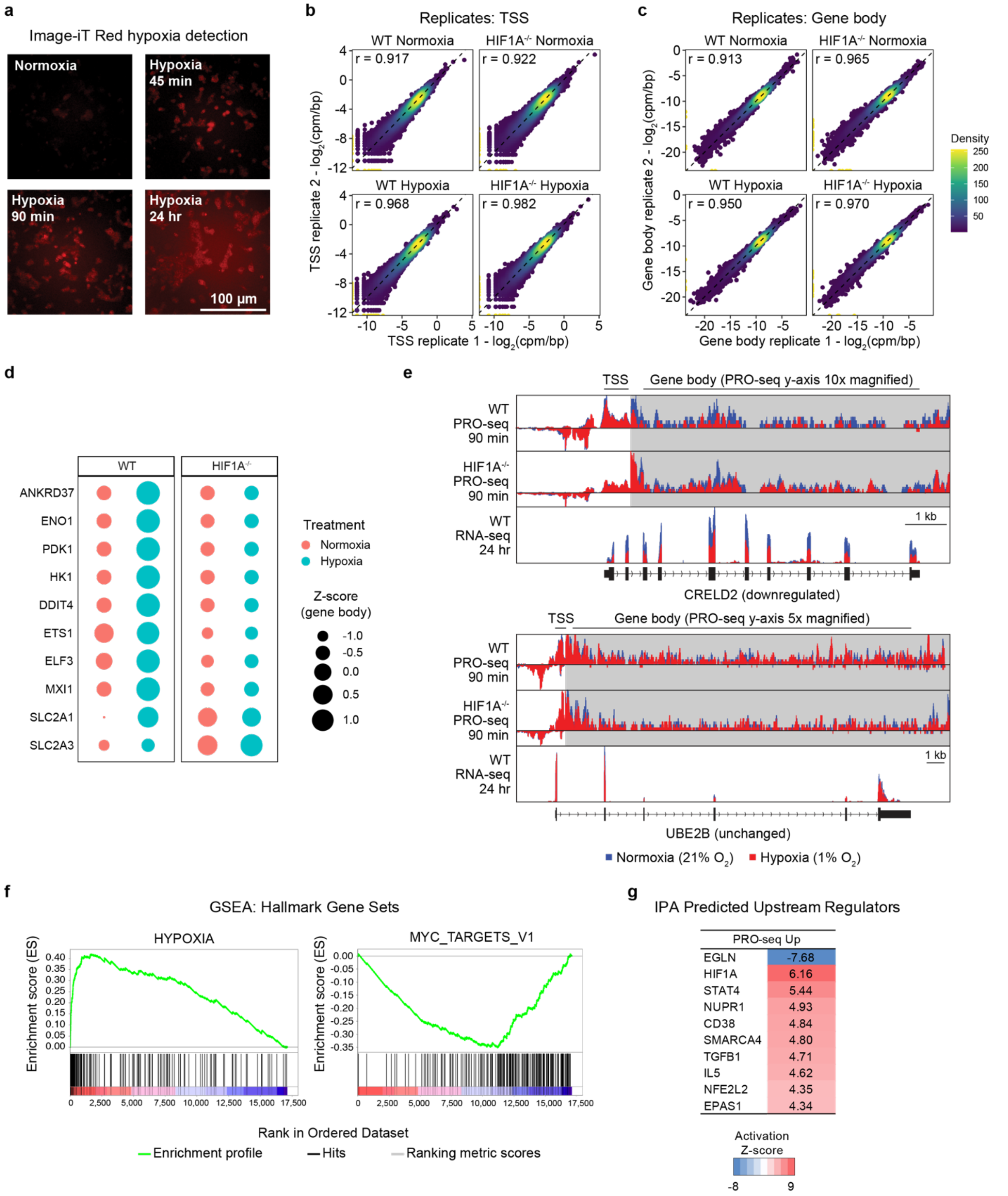

Supplementary Figure 1. Effect of acute hypoxia on transcription, related to Figure 1.
(**a**) Live-cell staining of HCT116 wild-type cells exposed to normoxia (21% O_2) or hypoxia (1% O_2) for the indicated times with the hypoxia-sensitive probe Image-iT Red. Images are representative of two replicates with similar results.

(b) Scatter plots comparing PRO-seq signal at transcription start site (TSS) regions of all genes for each pair of replicate samples. Numbers in upper left are Pearson correlation scores. Points are colored by density.
(c) Scatter plots comparing PRO-seq signal at gene body regions of all genes for each pair of replicate samples. Numbers in upper left are Pearson correlation scores. Points are colored by density.
(**d**) Bubble plots showing relative transcriptional activity for example genes in HCT116 wild-type (WT) and HIF1A$^{-/-}$ cells exposed to normoxia (pink) or hypoxia (teal). Circle areas correspond to gene-wise Z-scores calculated from gene body RPKM (reads per kilobase of transcript per million mapped reads) values.
(e) Genome browser views of PRO-seq and RNA-seq signals across the *CRELD2* and *UBE2B* loci for HCT116 WT and HIF1A$^{-/-}$ cells exposed to normoxia (blue) or hypoxia (red). Note that the y-axis for PRO-seq signal in gene bodies has been magnified to better allow visualization of this region.
(**f**) Gene set enrichment analysis (GSEA) plots for the Hypoxia and Myc Targets v1 Hallmark gene sets from MSigDB. Green lines indicate cumulative enrichment score; black bars indicate gene set hits among all genes ranked by $\log_2$-fold change (WT HCT116 hypoxia/normoxia).
(**g**) Heatmap of activation Z-scores for upstream regulator predictions by the Ingenuity Pathway Analysis suite for genes upregulated at 90 minutes hypoxia.

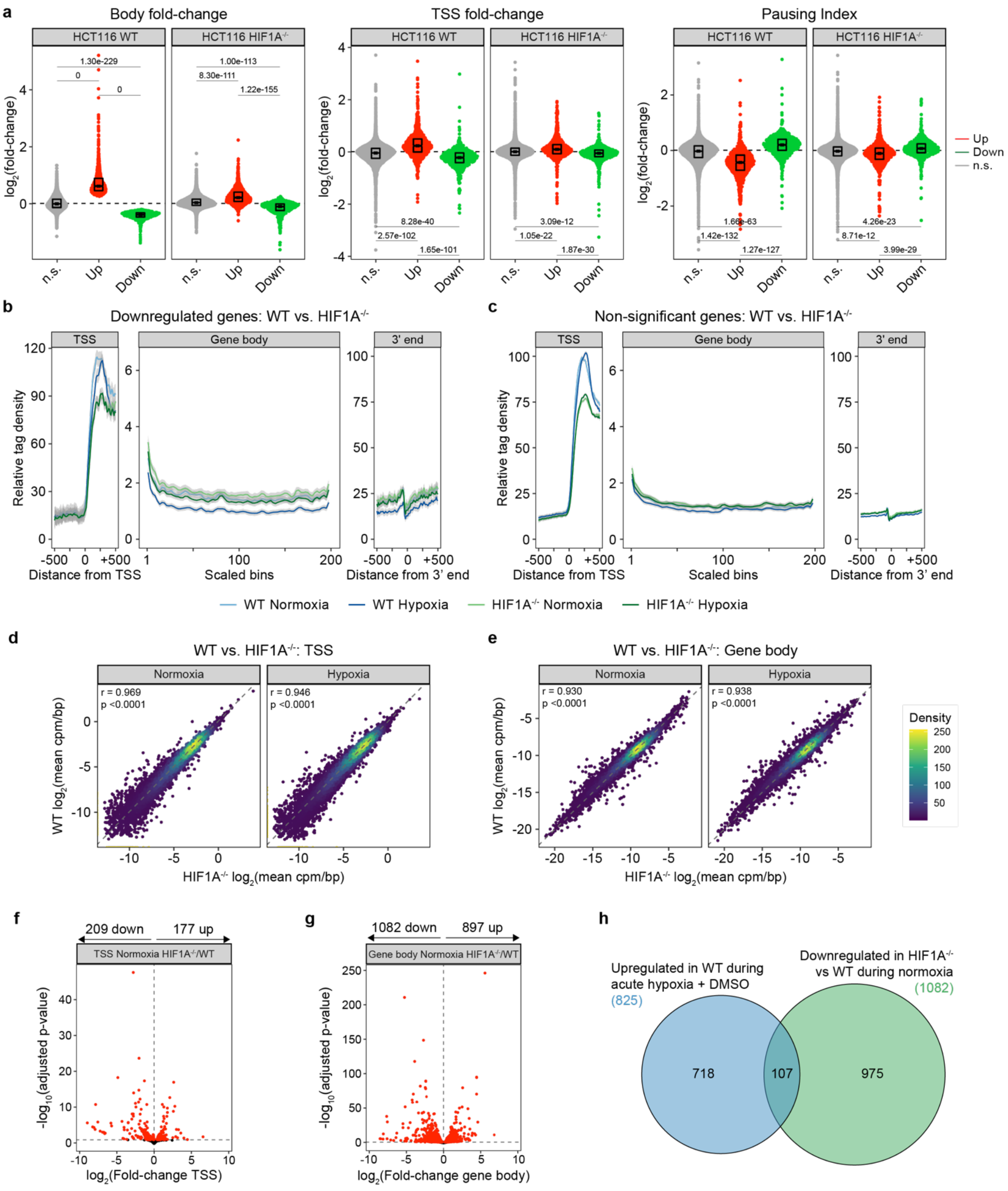

Supplementary Figure 2. Effect of acute hypoxia on transcription, related to Figure 1.
(**a**) Comparison of distributions of fold-changes in gene body and transcription start site (TSS) activity and pausing index for genes with no significant change (n.s., grey) in gene body activity compared to up- (red) and down-regulated (green) genes in HCT116 wild-type (WT) and HIF1A$^{-/-}$ cells. Horizontal spread of data points is proportional to density; boxes indicate medians and

upper and lower quartiles; horizontal bars with numbers indicate FDR-adjusted p-values for two-sided Mann-Whitney U tests.
(b) Metagene showing typical PRO-seq signal profiles across downregulated genes in HCT116 WT (blue) and HIF1A$^{-/-}$ (green). Data are represented as splined linear fit lines with 95% confidence intervals in grey.
(c) Metagene showing typical PRO-seq signal profiles across genes with no significant change in gene body activity in HCT116 WT (blue) and HIF1A$^{-/-}$ (green). Data are represented as splined linear fit lines with 95% confidence intervals in grey.
(**d**) Scatter plots comparing mean PRO-seq signal at TSS regions of all genes in HCT116 WT to HIF1A$^{-/-}$ cells exposed to normoxia (21% O_2) or hypoxia (1% O_2). Points are colored by density; numbers in upper left are Pearson correlations scores and corresponding p-values.
(e) Scatter plots comparing mean PRO-seq signal at gene body regions of all genes in HCT116 WT to HIF1A$^{-/-}$ cells exposed to normoxia (21% O_2) or hypoxia (1% O_2). Points are colored by density. Numbers in upper left are Pearson correlations scores and corresponding p-values.
(**f**) DESeq2 differential expression analysis of PRO-seq signal within TSS regions for HCT116 HIF1A$^{-/-}$ versus WT cells in normoxia. Horizontal dashed line indicates an FDR threshold of 10% for negative binomial Wald test; numbers above plot and colored points indicate significant genes at this threshold.

(g) DESeq2 differential expression analysis of PRO-seq signal within gene body regions for HCT116 HIF1A$^{-/-}$ versus WT cells in normoxia. Horizontal dashed line indicates an FDR threshold of 10% for negative binomial Wald test; numbers above plot and colored points indicate significant genes at this threshold.
(**h**) Venn diagram showing overlap between genes with upregulated gene body activity in WT cells during acute hypoxia and genes with downregulated gene body activity in HIF1A$^{-/-}$ cells (relative to WT) in normoxia.

Supplementary Fig. 3

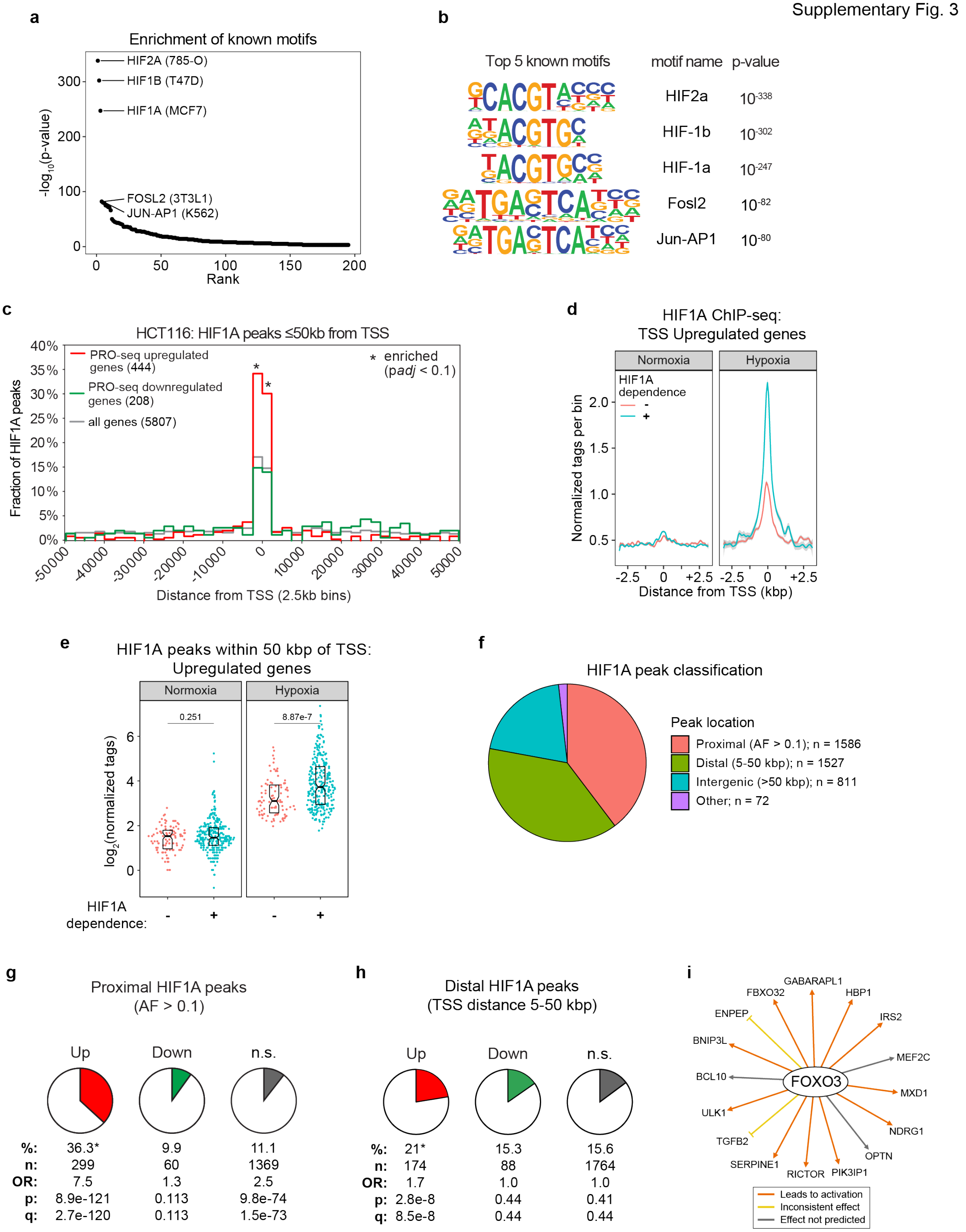

Supplementary Figure 3. Analysis of HIF1A chromatin binding in HCT116 cells, related to Figure 2.

(**a**) Comparison of p-value and rank for enrichment analysis of known sequence motifs underlying HIF1A peaks, using the binomial test in the HOMER software suite. Labels in parentheses indicate the cell line from which the motif was derived.
(**b**) Top 5 enriched motifs with p-values as in (a). Relative heights of letters represent position-specific probabilities for each base.
(**c**) Relative enrichment of HIF1A peaks in 2.5 kbp bins around transcription start sites (TSS) for genes sets defined by PRO-seq gene body differential expression class: all genes (gray), upregulated (red), downregulated (green). * indicates significant overrepresentation by two-sided hypergeometric test (Bonferroni adjusted p-values: bin -2.5 kbp = 1.10E-18, bin 0 kbp = 1.08E-16).
(d) Meta profile showing typical HIF1A occupancy profile over TSS of acute upregulated genes classified as HIF1A-independent (pink) or HIF1A-dependent (teal) in HCT116 cells exposed to normoxia or hypoxia. Data are represented as splined linear fit lines with 95% confidence intervals in grey.
(e) Distributions of HIF1A ChIP-seq enrichment signal (normalized tags per bin) for peaks within 50 kbp of TSS of upregulated genes, separated by dependence of upregulation on HIF1A: independent (pink) or dependent (teal). Horizontal spread of data points is proportional to density; boxes indicate medians and upper and lower quartiles; horizontal bars with numbers indicate p-values for two-sided Mann-Whitney U tests.
(**f**) Classification of HIF1A peaks identified in hypoxic HCT116 wild-type cells based on scoring by enrichment signal and distance from TSS.
(**g-h**) Proportions of genes in each PRO-seq gene body differential expression class associated with proximal (g) and distal (h) HIF1A peaks. The number and percentage of genes in each class associated with peaks of each type are listed below each pie chart. Odds ratios (OR), p-values, and q-values (Benjamini-Hochberg) are for two-sided Fisher's exact tests.
(**i**) FOXO3-regulated genes among acutely upregulated genes lacking associated HIF1A binding. Line colors indicate consistency of upregulation with known relationship types from the Ingenuity Pathway Analysis suite.

Supplementary Fig. 4

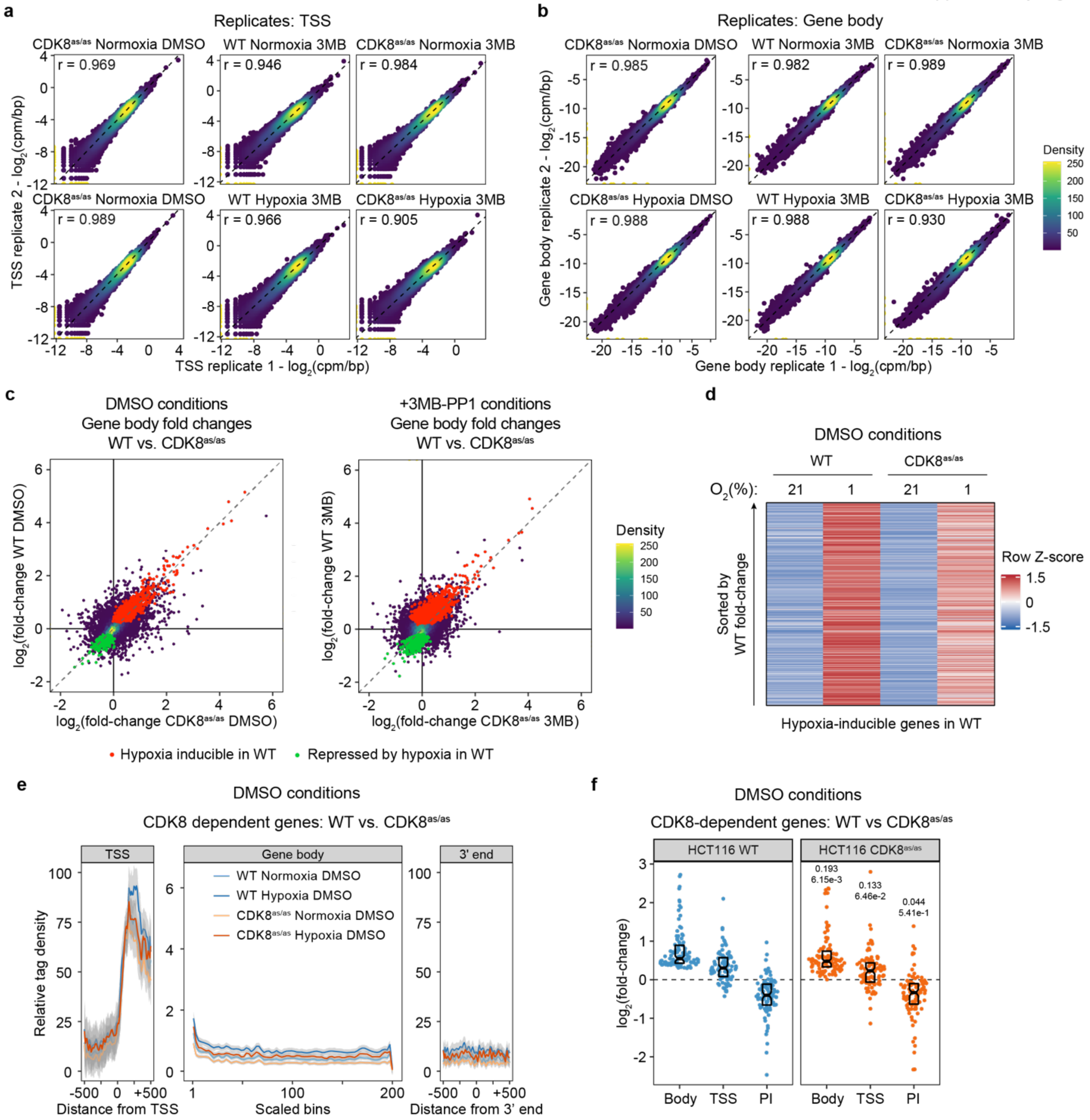

Supplementary Figure 4. Effect of CDK8 activity on transcription of hypoxia-inducible genes, related to Figure 3.

(a) Scatter plots comparing PRO-seq signal at TSS regions of all genes for each pair of replicate samples. Numbers in upper left are Pearson correlation scores. Points are colored by density.

(b) Scatter plots comparing PRO-seq signal at gene body regions of all genes for each pair of replicate samples. Numbers in upper left are Pearson correlation scores. Points are colored by density.

(**c**) Comparison of fold-changes in PRO-seq gene body signal induced by 90 minutes hypoxia in HCT116 wild-type (WT) versus CDK8$^{as/as}$ cells treated with DMSO (left) or 3MB-PP1 (right). Red/green points denote genes with significant up/down regulation in WT cells upon hypoxia; points representing all other genes are colored by density.

(**d**) Heatmap showing relative gene body signal for genes with increased transcription activity after 90 min hypoxia in WT and CDK8$^{as/as}$ HCT116 cells treated with DMSO during normoxia and hypoxia. Data are represented as row-wise Z-scores calculated from RPKM values.

(**e**) Metagene showing typical PRO-seq signal profiles across CDK8-dependent upregulated genes in HCT116 WT (blue) and CDK8$^{as/as}$ (orange) cells treated with DMSO. Data are represented as splined linear fit lines with 95% confidence intervals in grey.

(**f**) PRO-seq fold-change distributions for gene body, transcription start site (TSS) and pausing index (PI) of CDK8-dependent upregulated genes in HCT116 WT (blue) and CDK8$^{as/as}$ (orange) cells treated with DMSO. Horizontal spread of data points is proportional to density; boxes indicate medians and upper and lower quartiles. Numbers above the CDK8$^{as/as}$ plots indicate effect size estimates and p-values for two-sided Mann-Whitney U tests against the corresponding measure in WT cells.

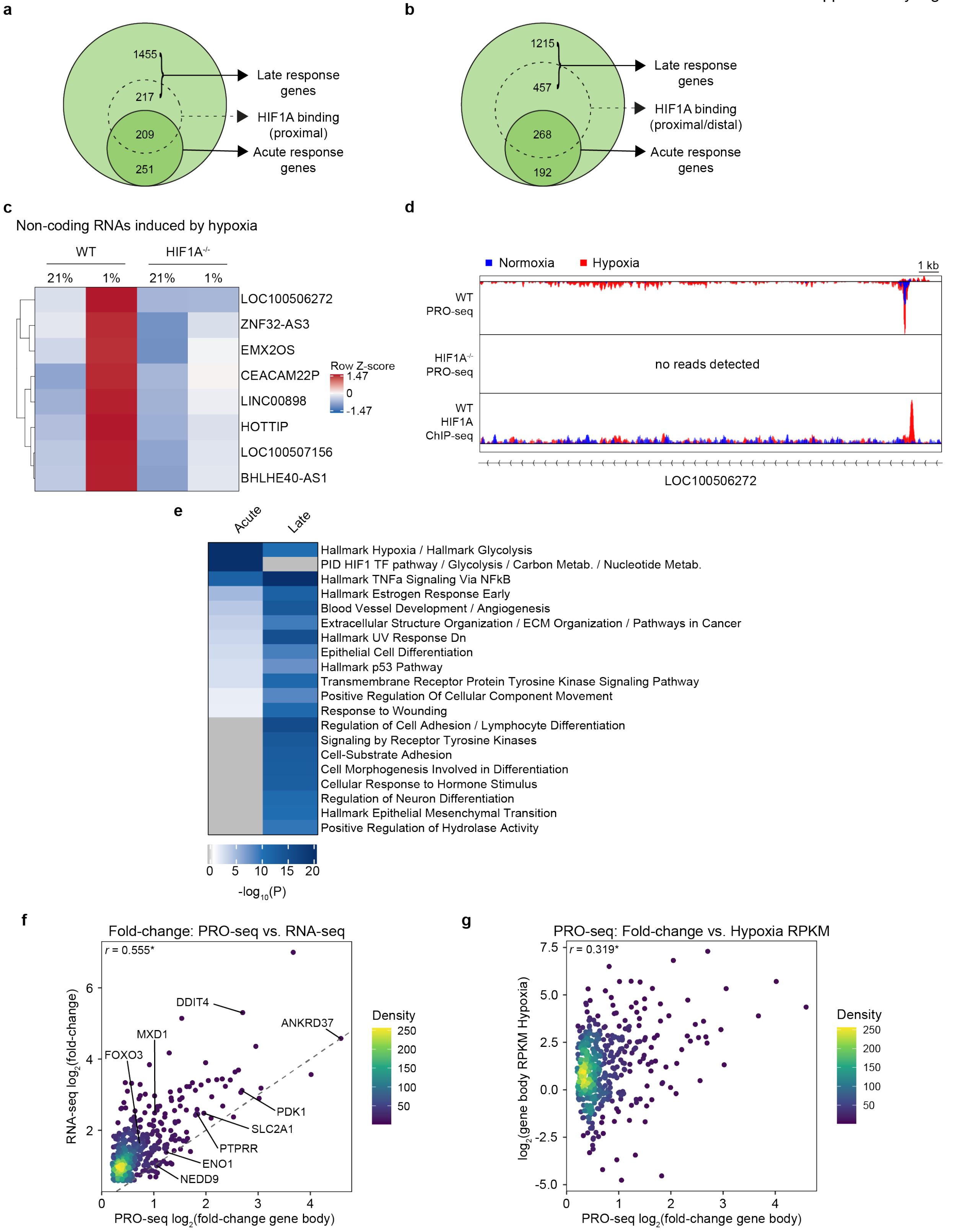

Supplementary Figure 5. Interplay between early transcriptional output, late steady-state mRNA levels, and chromatin landscape, related to Figure 4.

(**a-b**) Venn diagrams showing the proportions of acute response genes and late response genes associated with proximal and/or distal HIF1A binding sites.
(**c**) Heatmap showing relative gene body signal for non-coding RNA genes with increased transcription activity after 90 min in HCT116 wild-type (WT) and HIF1A$^{-/-}$ cells. Data are represented as row-wise Z-scores calculated from RPKM values.
(**d**) Genome browser view of PRO-seq and ChIP-seq signals across the *LOC100506272* locus for HCT116 WT and HIF1A$^{-/-}$ cells exposed to normoxia (blue) or hypoxia (1% O_2, red).

(**e**) Comparison of top 20 pathway/function clusters enriched among acute response genes and late response genes, as identified by Metascape. Heatmap color represents $-\log_{10}$(p-value) from hypergeometric enrichment test.
(**f**) Comparison of fold-changes in transcription activity after 90 minutes hypoxia and steady-state mRNA changes after 24 hrs hypoxia for acute response genes. Points are colored by density and selected example genes are labelled. Pearson correlation coefficient is shown in upper left, * denotes significant correlation ($p < 2.2e-16$).
(**g**) Comparison of fold-changes in transcription activity (gene body fold-change) with transcriptional output (gene body RPKM) after 90 minutes hypoxia for acute response genes. Points are colored by density and selected example genes are labelled. Pearson correlation coefficient is shown in upper left, * denotes significant correlation ($p = 2.38e-12$).

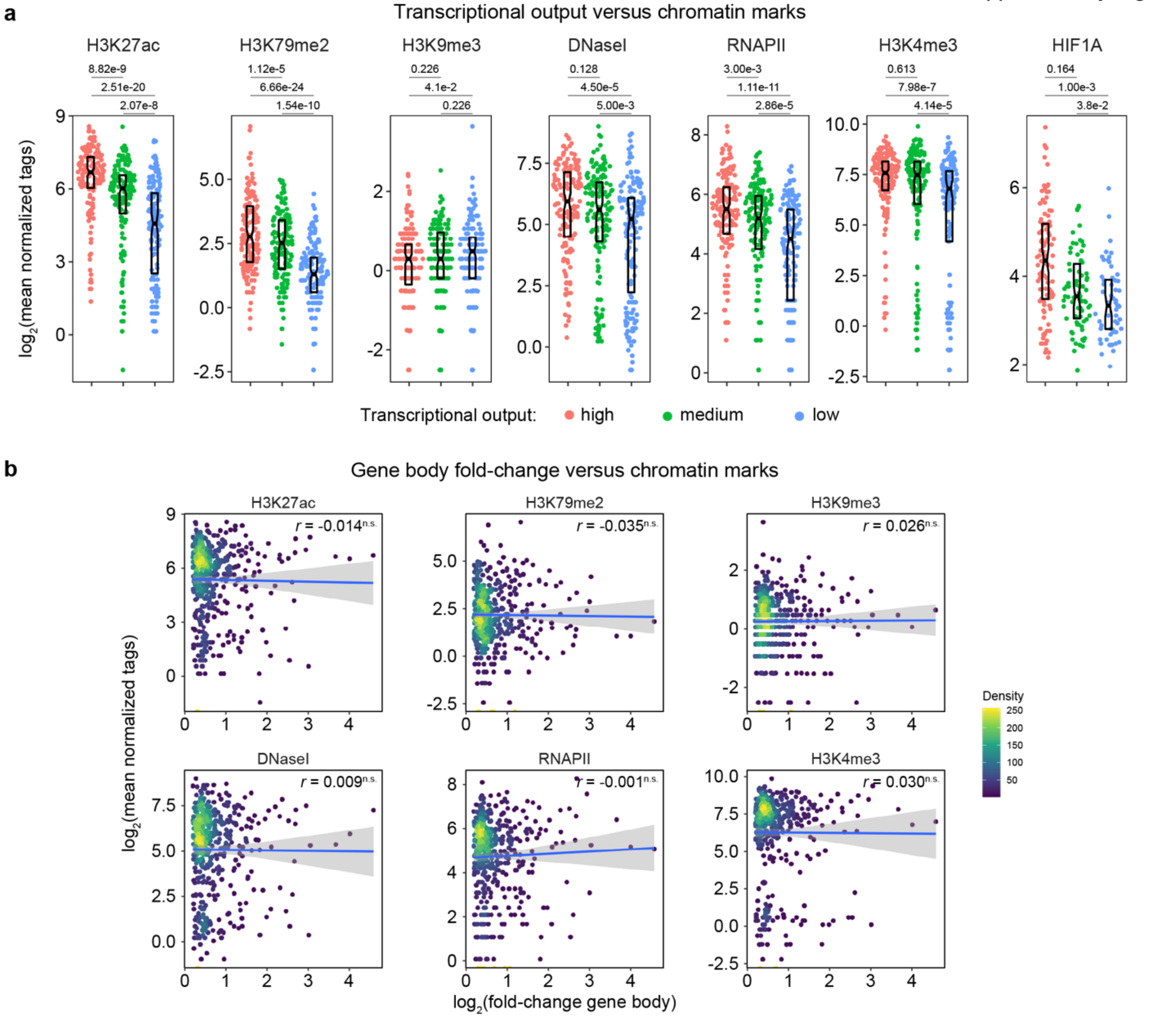

Supplementary Figure 6. Interplay between early transcriptional output, late steady-state mRNA levels, and chromatin landscape, related to Figure 4.

(**a**) Fold-change distributions for enrichment signal (mean normalized tag counts) at TSS regions (for ENCODE chromatin and RNAPII data) or peak regions (for HIF1A ChIP-seq) for acute response genes, separated into high (pink), medium (green), and low (blue) tertiles on the basis of gene body transcription activity at 90 minutes hypoxia. Horizontal spread of data points is proportional to density; boxes indicate medians and upper and lower quartiles. Horizontal bars with numbers above plots indicate FDR-adjusted p-values for two-sided Mann-Whitney U tests.

(**b**) Comparison of changes in transcription activity (gene body fold-change) after 90 minutes hypoxia with enrichment signal (mean normalized tag counts) at TSS regions for acute response genes. Points are colored by density.; blue lines denote linear model fits to the data, with 95% confidence intervals in grey. Numbers in upper left of each plot are Pearson correlation coefficients, n.s. denotes non-significant correlations (10% FDR).

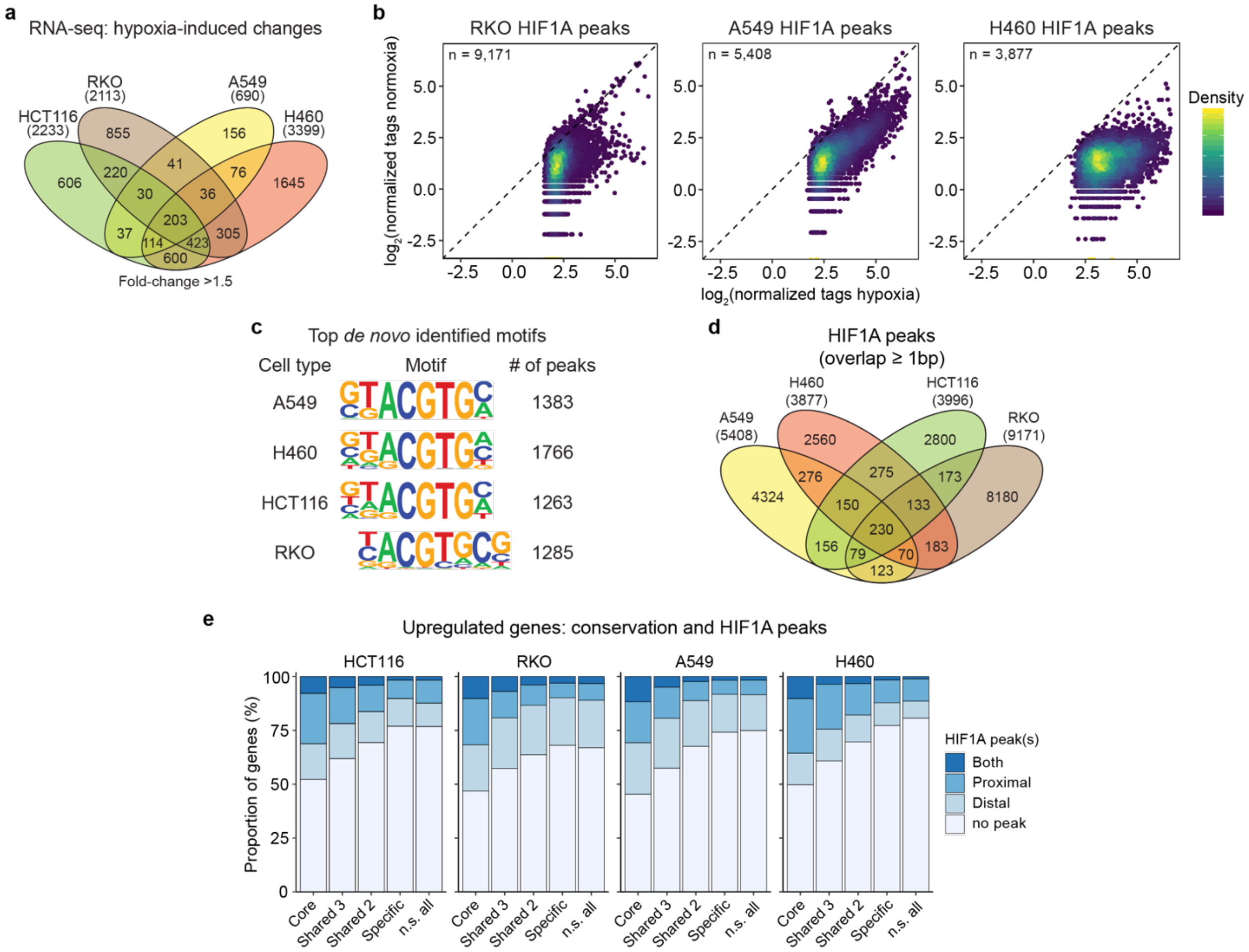

Supplementary Figure 7. Conservation of the acute transcriptional response to hypoxia is associated with the strength of HIF1A binding, related to Figure 5.

(**a**) Qualitative overlaps between genes called as upregulated (DESeq10% FDR, fold-change >1.5) in RNA-seq datasets for HCT116, RKO, A549, and H460 cells after 24 hr hypoxia (1% O_2).

(**b**) Comparison of enrichment signal (normalized tag counts) in normoxia and hypoxia for HIF1A peaks called in hypoxic RKO, A549, and H460 cells. Points are colored by density; numbers in upper left indicate number of detected peaks.

(**c**) Top enriched motifs identified in each cell line by enrichment analysis of *de novo* sequence motifs underlying HIF1A peaks using HOMER. Relative heights of letters represent position-specific probabilities for each base.

(**d**) Qualitative overlaps (≥ 1 bp) for HIF1A peaks called in hypoxic HCT116, RKO, A549, and H460 cells.

(**e**) Proportions of upregulated genes, as in (a), associated with proximal and/or distal HIF1A peaks in hypoxic HCT116, RKO, A549, and H460 cells, separated by gene class.

a

Significant correlations with HIF1A

Gene	rho	p	q (BH)
**DDIT4**	**0.30**	**4.60e-14**	**8.43e-10**
ARNT	0.17	2.41e-05	5.05e-02
**FOXO3**	**0.15**	**1.04e-04**	**7.72e-02**
**MPI**	**0.15**	**1.74e-04**	**8.88e-02**
**EGLN1**	**-0.22**	**1.96e-08**	**8.98e-05**
VHL	-0.25	3.60e-10	2.20e-06
HIF1AN	-0.26	4.05e-11	3.71e-07

b

Significant correlations with VHL

Gene	rho	p	q (BH)
ELOB	0.50	0	0
**EGLN1**	**0.40**	**0**	**0**
CUL2	0.33	0	0
**EGLN3**	**0.13**	**1.12e-03**	**6.13e-02**
**SLC2A1**	**0.13**	**1.34e-03**	**6.67e-02**
**DDIT4**	**-0.17**	**1.94e-05**	**6.47e-03**
NEDD8	-0.22	3.86e-08	6.43e-05
**HIF1A**	**-0.25**	**3.60e-10**	**9.44e-07**

c

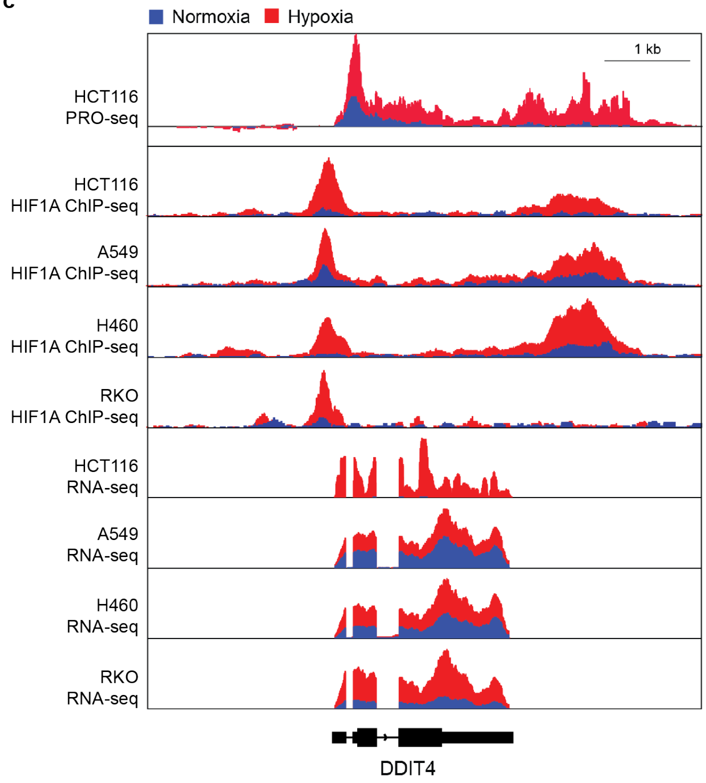

d

Significant correlations with DDIT4

Gene	rho	p	q (BH)
TSC1	0.43	0	0
TSC2	0.43	0	0
**HIF1A**	**0.30**	**4.60e-14**	**1.69e-10**
**FOXO3**	**0.15**	**1.18e-04**	**2.00e-02**
**GPI**	**-0.15**	**1.91e-04**	**2.78e-02**
VHL	-0.17	1.94e-05	7.72e-03
LAMTOR1	-0.24	2.57e-09	4.72e-06
MTOR	-0.25	1.16e-10	2.66e-07

Supplementary Figure 8. Genetic screening reveals a tumor suppressive role for HIF1A linked to DDIT4 and mTOR suppression, related to Figure 6.
(**a**) Summary of significant Spearman correlations with *HIF1A* with acute response genes in bold. Q values were calculated with the Benjamini-Hochberg adjustment method (BH).
(**b**) Summary of significant Spearman correlations with *VHL* with acute response genes in bold. Q values were calculated with the Benjamini-Hochberg adjustment method (BH).
(**c**) PRO-seq (HCT116 only), ChIP-seq, and RNA-seq signals across the *DDIT4* locus for HCT116, RKO, A549, and H460 cells exposed to normoxia (blue) or hypoxia (24 hrs 1% O_2, red).
(**d**) Summary of significant Spearman correlations with *DDIT4* with acute response genes in bold. Q values were calculated with the Benjamini-Hochberg adjustment method (BH).

Supplementary Fig. 9

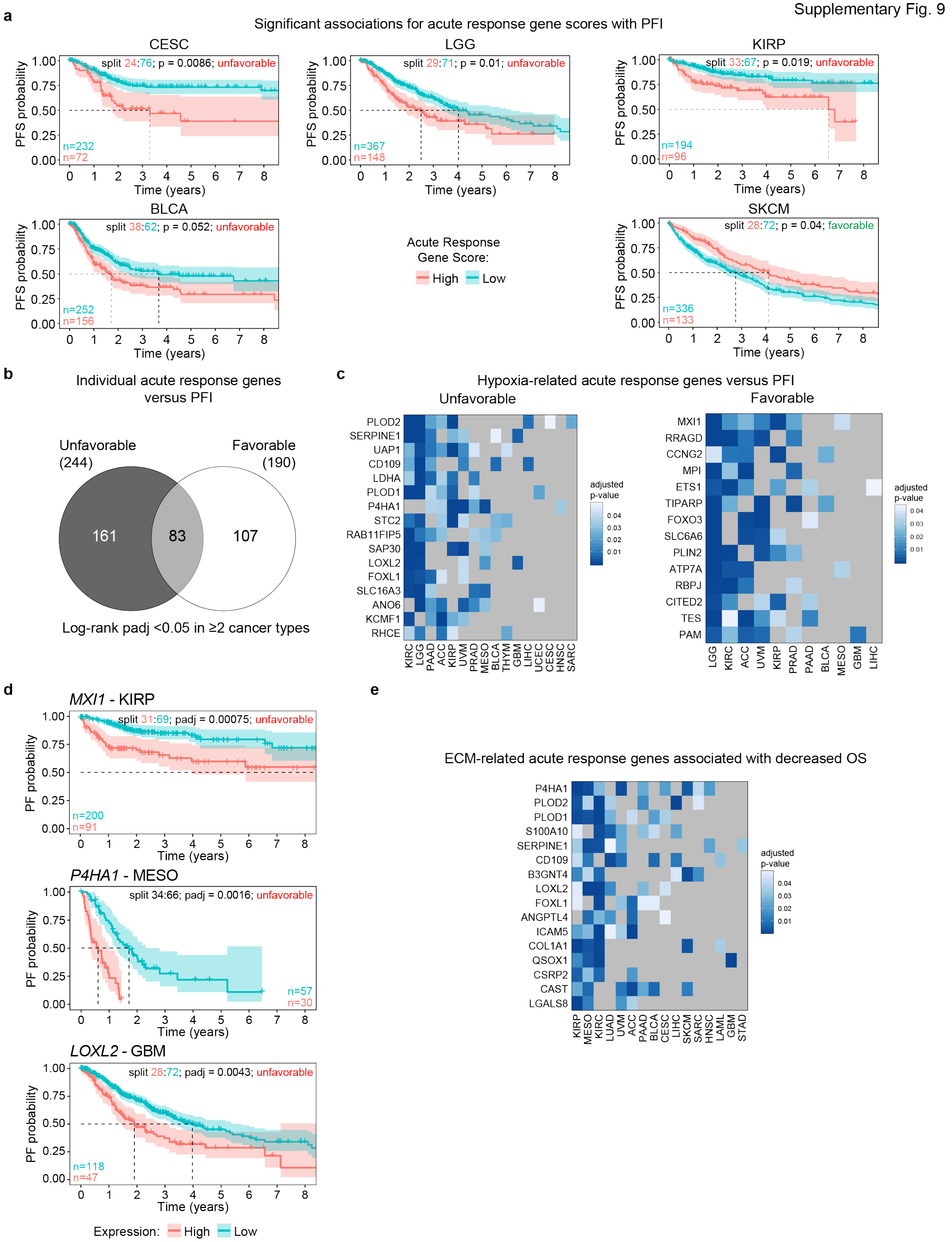

Supplementary Figure 9. Expression of HIF1A targets involved in ECM remodeling associates with poor prognosis across multiple cancer types, Related to Figure 7.

(**a**) Kaplan-Meier plots for progression-free interval (PFI) in the indicated cancer types, separated by Acute Response Gene Score; split indicates the proportions of samples placed into high and low scoring groups, with initial numbers at risk per arm indicated at lower left. P-values are from log-rank analysis with the indicated split.
(**b**) Overlap between significant (FDR 10%) unfavorable genes in iterative log-rank and Cox regression analyses. See Supplementary Data 8 for exact FDR values.
(**c**) Heatmaps showing significant FDR-adjusted p-values for log-rank analysis of PFI across cancer types for unfavorable and favorable hypoxia-related genes.
(**d**) Kaplan-Meier plots for selected individual genes in the indicated cancer types, separated by expression level; split indicates the proportions of samples placed into high and low expression groups, with initial numbers at risk per arm indicated at lower left or right. P-values are from log-rank analysis with the indicated split.
(**e**) Heatmap showing significant FDR-adjusted p-values for log-rank analysis across cancer types for ECM-related genes associated with lower overall survival (OS).
