# Supplementary material for: Multi-omics analysis reveals contextual tumor suppressive and oncogenic gene modules within the acute hypoxic response
Source: Nat Commun. 2021 Mar 2;12:1375. doi: 10.1038/s41467-021-21687-2 (PMC7925689; doi:10.1038/s41467-021-21687-2)
Supplement: Supplementary file 3 — Description of Additional Supplementary Files [file 41467_2021_21687_MOESM3_ESM.pdf]

Description of Additional Supplementary Files

Supplementary Data 1 – PROseq results WT versus HIF1A null cells.

A. Tab "DE Body WT Norm|Hypox" - PRO-seq gene body DESeq2 negative binomial Wald test results for HCT116 wild-type cells (90 min hypoxia vs. Normoxia).

B. Tab "DE TSS WT Norm|Hypox" - PRO-seq transcription start site DESeq2 negative binomial Wald test results for HCT116 wild-type cells (90 min hypoxia vs. Normoxia).

C. Tab "DE Body HIF1Ako Norm|Hypox" - PRO-seq gene body DESeq2 negative binomial Wald test results for HCT116 HIF1A$^{-/-}$ cells (90 min hypoxia vs. Normoxia).

D. Tab "DE TSS HIF1Ako Norm|Hypox" - PRO-seq transcription start site DESeq2 negative binomial Wald test results for HCT116 HIF1A-/- cells (90 min hypoxia vs. Normoxia).

E. Tab "DE TSS Norm WT|HIF1Ako" - PRO-seq transcription start site DESeq2 negative binomial Wald test results for HCT116 wild-type (normoxia) vs. HCT116 HIF1A$^{-/-}$ cells (normoxia).

F. Tab "DE Body Norm WT|HIF1Ako" - PRO-seq gene body DESeq2 negative binomial Wald test results for HCT116 wild-type (normoxia) vs. HCT116 HIF1A$^{-/-}$ cells (normoxia).

G. Tab "DE Body Hypox WT|HIF1Ako" - PRO-seq gene body DESeq2 negative binomial Wald test results for HCT116 wild-type (90 min hypoxia) vs. HCT116 HIF1A$^{-/-}$ (90 min hypoxia).

H. Tab "WT Pausing" - PRO-seq pausing analysis results for HCT116 wild-type (normoxia and 90 min hypoxia).

I. Tab "HIF1Ako Pausing" - PRO-seq pausing analysis results for HCT116 HIF1A-/- (normoxia and 90 min hypoxia).

Supplementary Data 2 – Hypoxia Inducible Genes by PRO-seq - 825 genes - Genes induced in response to acute hypoxia (PRO-seq upregulation at 90 min) in HCT116 wild-type cells.

Supplementary Data 3 – HIF1A ChIPseq.

A. Tab "HCT116 peaks" - ChIP-seq: HIF1A peaks identified in HCT116 cells exposed to hypoxia (1% O2) for 24 hours.

B. Tab "HCT116 assoc genes" - ChIP-seq: genes associated with proximal and distal HIF1A peaks identified in HCT116 cells exposed to hypoxia (1% O2) for 24 hours.

C. Tab "RKO peaks" - ChIP-seq: HIF1A peaks identified in RKO cells exposed to hypoxia (1% O2) for 24 hours.

D. Tab "RKO assoc genes" - ChIP-seq: genes associated with proximal and distal HIF1A peaks identified in RKO cells exposed to hypoxia (1% O2) for 24 hours.

E. Tab "A549 peaks" - ChIP-seq: HIF1A peaks identified in A549 cells exposed to hypoxia (1% O2) for 24 hours.

F. Tab "A549 assoc genes" - ChIP-seq: genes associated with proximal and distal HIF1A peaks identified in A549 cells exposed to hypoxia (1% O2) for 24 hours.

G. Tab "H460 peaks" - ChIP-seq: HIF1A peaks identified in H460 cells exposed to hypoxia (1% O2) for 24 hours.

H. Tab "H460 assoc genes" - ChIP-seq: genes associated with proximal and distal HIF1A peaks identified in H460 cells exposed to hypoxia (1% O2) for 24 hours.

Supplementary Data 4. PROseq results WT CDK8as DMSO 3MB.

A. Tab “DE Body CDK8as Norm|Hypox” - PRO-seq gene body DESeq2 negative binomial Wald test results for HCT116 CDK8as/as DMSO (90 min hypoxia vs. normoxia).

B. Tab “DE Body Norm WT|CDK8as” - PRO-seq gene body DESeq2 negative binomial Wald test results for HCT116 CDK8as/as (DMSO normoxia) vs. HCT116 wild-type cells (DMSO normoxia).

C. Tab “DE Body WT 3MB Norm|Hypox” - PRO-seq gene body DESeq2 negative binomial Wald test results for HCT116 wild-type cells treated with 3MB-PP1 (90 min hypoxia vs. Normoxia).

D. Tab “DE Body CDK8as 3MB Norm|Hypox” - PRO-seq gene body DESeq2 negative binomial Wald test results for HCT116 CDK8as/as cells treated with 3MB-PP1 (90 min hypoxia vs. normoxia).

E. Tab “DE Body 3MB Hypox WT|CDK8as” - PRO-seq gene body DESeq2 negative binomial Wald test results for HCT116 CDK8as/as cells treated with 3MB-PP1 and hypoxia (3MB Hypoxia) vs. HCT116 wild-type cells treated with 3MB-PP1 and hypoxia (3MB Hypoxia).

F. Tab “CDK8 DMSO Pausing” - PRO-seq pausing analysis results for HCT116 CDK8as/as cells treated with DMSO (Normoxia and 90 min Hypoxia).

G. Tab “WT 3MB Pausing” - PRO-seq pausing analysis results for HCT116 wild-type cells treated with 3MB-PP1 (normoxia and 90 min hypoxia).

H. Tab “CDK8 3MB Pausing” - PRO-seq pausing analysis results for HCT116 CDK8as/as cells treated with 3MB-PP1 (Normoxia and 90 min Hypoxia).

Supplementary Data 5 – RNAseq results.

A. Tab “HCT116” - DESeq2 negative binomial Wald test results for HCT116 wild-type cells (24hr Hypoxia vs. Normoxia).

B. Tab “HCT116 HIF1Ako” - DESeq2 negative binomial Wald test results for HCT116 HIF1A-/- cells (24hr Hypoxia vs. Normoxia).

C. Tab “RKO” - DESeq2 negative binomial Wald test results for RKO wild-type cells (24hr Hypoxia vs. Normoxia).

D. Tab “A549” - DESeq2 negative binomial Wald test results for A549 wild-type cells (24hr Hypoxia vs. Normoxia).

E. Tab “H460” - DESeq2 negative binomial Wald test results for H460 wild-type cells (24hr Hypoxia vs. Normoxia).

Supplementary Data 6 – Hypoxia-inducible ncRNAs.

Tab “Hypoxia-inducible ncRNAs” - PRO-seq gene body DESeq2 negative binomial Wald test results for HCT116 wild-type cells (90 min hypoxia vs. Normoxia) for Hypoxia-inducible ncRNA genes.

Supplementary Data 7 – DepMap co-dependency analysis.

A. Tab “HIF1A” - Spearman correlation analysis and significance results for DepMap HIF1A gene effect.

B. Tab “VHL” - Spearman correlation analysis and significance results for DepMap VHL gene effect.

C. Tab “DDIT4” - Spearman correlation analysis and significance results for DepMap DDIT4 gene effect.

Supplementary Data 8 – TCGA survival analysis.

A. Tab “LogRank Hypoxia Score vs. PFI” - Iterative log-rank results for Acute Hypoxia Score vs. progression-free interval (PFI).

B. Tab “LogRank Gene Exp. vs. PFI” - Iterative log-rank results for individual gene expression vs. progression-free interval (PFI).

C. Tab “LogRank Gene Exp. Vs. OS’ - Iterative log-rank results for individual gene expression vs. overall survival (OS).

D. Tab “Cox Gene Expression vs. PFI” - Cox regression results for individual gene expression vs. progression-free interval (PFI).
