# Supplementary material for: Multi-omics analysis reveals contextual tumor suppressive and oncogenic gene modules within the acute hypoxic response
Source: Nat Commun. 2021 Mar 2;12:1375. doi: 10.1038/s41467-021-21687-2 (PMC7925689; doi:10.1038/s41467-021-21687-2)
Supplement: Supplementary file 12 — Reporting Summary [file 41467_2021_21687_MOESM12_ESM.pdf]

nature research

Corresponding author(s): Matthew D. Galbraith, Joaquin M. Espinosa

Last updated by author(s): Jan 26, 2021

Reporting Summary

Nature Research wishes to improve the reproducibility of the work that we publish. This form provides structure for consistency and transparency in reporting. For further information on Nature Research policies, see our Editorial Policies and the Editorial Policy Checklist.

Statistics

For all statistical analyses, confirm that the following items are present in the figure legend, table legend, main text, or Methods section.

n/a	Confirmed	
☐	☒	The exact sample size (*n*) for each experimental group/condition, given as a discrete number and unit of measurement
☐	☒	A statement on whether measurements were taken from distinct samples or whether the same sample was measured repeatedly
☐	☒	The statistical test(s) used AND whether they are one- or two-sided<br>*Only common tests should be described solely by name; describe more complex techniques in the Methods section.*
☐	☒	A description of all covariates tested
☐	☒	A description of any assumptions or corrections, such as tests of normality and adjustment for multiple comparisons
☐	☒	A full description of the statistical parameters including central tendency (e.g. means) or other basic estimates (e.g. regression coefficient) AND variation (e.g. standard deviation) or associated estimates of uncertainty (e.g. confidence intervals)
☐	☒	For null hypothesis testing, the test statistic (e.g. *F*, *t*, *r*) with confidence intervals, effect sizes, degrees of freedom and *P* value noted<br>*Give P values as exact values whenever suitable.*
☒	☐	For Bayesian analysis, information on the choice of priors and Markov chain Monte Carlo settings
☒	☐	For hierarchical and complex designs, identification of the appropriate level for tests and full reporting of outcomes
☐	☒	Estimates of effect sizes (e.g. Cohen's *d*, Pearson's *r*), indicating how they were calculated

Our web collection on statistics for biologists contains articles on many of the points above.

Software and code

Policy information about availability of computer code

Data collection	PRO-seq data were collected using the NextSeq 500 platform (Illumina), ChIP-seq data were collected using the HiSeq 4000 platform (Illumina), and RNA-seq data for RKO, A549, and H460 cell lines were collected using the HiSeq 4000 platform (Illumina). Western Blot images were captured on an ImageQuant LAS4000 (GE). Live cell fluorescence images were captured using an Olympus IX71 microscope. Sources for all other data are described in the main text and/or methods.
Data analysis	Preprocessing and primary analysis of sequencing data was carried out using FASTQC (v0.11.2, v0.11.5), FastQ Screen (v0.4.4, v0.11.0), BBDUK (BBTools, v37.99), FASTQ-MCF (EAUtils, v1.05), Hisat2 (v2.1.0), Samtools (v1.5, v1.19), featureCounts (Subread package, v1.6.2), DESeq2 (v1.6.3, v1.22.1), HOMER (v4.9.1, v4.3), IGV (v2.8), Fastx toolkit (v0.0.13.2), TopHat2 (v2.0.13), Picard (v1.129), RSeQC (v2.6), HTSeq (v0.6.1), Bowtie2 (v2.2.9), MAnorm (v1.1.4), as described in the main text and methods. Secondary and statistical analyses were carried out using R (v3.5.1) / RStudio (v1.1.453) with the following packages: Bioconductor v3.7, DESeq2 (v1.22.1), ggplot2 (v3.1.0), GenomicRanges (v1.36.0), Gviz (v1.26.4), survminer (v0.4.6), survival (v2.44-1.1), purrr (0.3.3), finalfit (0.9.5), furrr (0.1.0), as described in the main text and methods. Pathway/function enrichment analyses were carried out using the GSEA preranked module (August 2019, https://cloud.genepattern.org), the Upstream Regulator Analysis module within the Ingenuity Pathway Analysis (IPA) suite (Fall 2019 Release, http://www.ingenuity.com), and Metascape (3.5, https://metascape.org).

For manuscripts utilizing custom algorithms or software that are central to the research but not yet described in published literature, software must be made available to editors and reviewers. We strongly encourage code deposition in a community repository (e.g. GitHub). See the Nature Research guidelines for submitting code & software for further information.

Data

Policy information about availability of data

All manuscripts must include a data availability statement. This statement should provide the following information, where applicable:

- Accession codes, unique identifiers, or web links for publicly available datasets
- A list of figures that have associated raw data
- A description of any restrictions on data availability

Raw data generated during this study are available at Gene Expression Omnibus database under accessions GSE145567 (PRO-seq), GSE145157 (HIF1A ChIP-seq data for HCT116, RKO, A549, and H460), GSE145108 (RNA-seq for RKO, A549, H460), and GSE68297 (RNA-seq for HCT116). Processed data are included as Supplementary Excel Tables.

Field-specific reporting

Please select the one below that is the best fit for your research. If you are not sure, read the appropriate sections before making your selection.

☒ Life sciences ☐ Behavioural & social sciences ☐ Ecological, evolutionary & environmental sciences

For a reference copy of the document with all sections, see nature.com/documents/nr-reporting-summary-flat.pdf

Life sciences study design

All studies must disclose on these points even when the disclosure is negative.

Sample size	No sample size calculations were performed. Sample size was determined to be adequate based on consistency of replicates and measurable differences between groups.
Data exclusions	No data were excluded from analysis.
Replication	All PRO-seq, ChIP-seq, and RNA-seq data were generated from independent biological duplicates. Western blot and fluorescence images are representative examples of multiple replicates.
Randomization	Sample randomization was not carried out as this was not necessary due to the isogenic nature of cell lines being compared within each treatment and control group.
Blinding	Binding was not carried out during sample collection and processing. All data analysis of sequencing data was carried out in an automated fashion without exclusion of any samples.

Reporting for specific materials, systems and methods

We require information from authors about some types of materials, experimental systems and methods used in many studies. Here, indicate whether each material, system or method listed is relevant to your study. If you are not sure if a list item applies to your research, read the appropriate section before selecting a response.

Materials & experimental systems

n/a	Involved in the study
☐	☒ Antibodies
☐	☒ Eukaryotic cell lines
☒	☐ Palaeontology and archaeology
☒	☐ Animals and other organisms
☒	☐ Human research participants
☒	☐ Clinical data
☒	☐ Dual use research of concern

Methods

n/a	Involved in the study
☐	☒ ChIP-seq
☒	☐ Flow cytometry
☒	☐ MRI-based neuroimaging

Antibodies

Antibodies used	HIF1A (western blot) BD Biosciences BDB610959; lot 8025665; used at 1:1,500 dilution.<br>HIF1A (ChIP-seq) Novus Biologicals NB100-134; lot AE-3<br>HIF2A Cell Signaling Technology 59973; lot 2; used at 1:1,000 dilution.<br>alpha-tubulin Sigma T9026; lot unknown; used at 1:10,000 dilution.<br>Goat Anti-Rabbit IgG (H + L)-HRP Conjugate BioRad 1706515; lot L005679A<br>Immun-Star Goat Anti-Mouse (GAM)-HRP Conjugate BioRad 1705047; lot L005662D
Validation	Anti-HIF1A antibody used for ChIP-seq (Novus Biologicals NB100-134, lot AE-3) was validated both by knock out and by ChIP qPCR

Validation	using established HIF1A binding loci. Anti-HIF1A antibody used for western blots was validated by knock-out, detected protein size, and inducibility upon hypoxia. Anti-HIF2A antibody specificity was validated by detected protein size and inducibility upon hypoxia. Validation of anti-alpha-tubulin and HRP-conjugated secondary antibodies was based on manufacturer information.

Eukaryotic cell lines

Policy information about cell lines

Cell line source(s)	HCT116 (CCL-247), RKO (CLR-2577), A549 (CCL-185), and H460 (HTB-177) cell lines were obtained from ATCC. HCT116 HIF1A-/- cells (Dang et al., 2006) were a generous gift from Dang lab. HCT116 CDK8as/as cells were engineered as described previously (Galbraith et al., Cell Reports 2017).
Authentication	All cell lines used in this study were authenticated using STR profiling.
Mycoplasma contamination	All lines tested negative for mycoplasma contamination using PCR as published by Timenetsky et al. in 2006.
Commonly misidentified lines (See ICLAC register)	No commonly misidentified cell lines were used in this study.

ChIP-seq

Data deposition

☒ Confirm that both raw and final processed data have been deposited in a public database such as GEO.

☒ Confirm that you have deposited or provided access to graph files (e.g. BED files) for the called peaks.

Data access links<br>*May remain private before publication.*	PRO-seq: https://www.ncbi.nlm.nih.gov/geo/query/acc.cgi?acc=GSE145567<br>ChIP-seq: https://www.ncbi.nlm.nih.gov/geo/query/acc.cgi?acc=GSE145157<br>RNA-seq: https://www.ncbi.nlm.nih.gov/geo/query/acc.cgi?acc=GSE145108
Files in database submission	Raw:<br>FASTQ files for all PRO-seq, ChIP-seq, and RNA-seq samples<br>Processed:<br>Count tables for PRO-seq TSS and Gene body regions.<br>HOMER peak calling outputs for HIF1A ChIP-seq.<br>BedGraph files for all PRO-seq and ChIP-seq samples.<br>Gene-level count and RPKM tables for RNA-seq samples.
Genome browser session (e.g. UCSC)	no longer applicable

Methodology

Replicates	Two independent replicates prepared from cells exposed to either normoxia and hypoxia were used for immunoprecipitation, library preparation and sequenced. To increase reliability of identified HIF1A REs both replicates were combined prior to peak calling.
Sequencing depth	ChIP-seq data yield was ~52-93 x 10^6 raw 150 bp single-end reads and ~19-46 x 10^6 final mapped reads per sample
Antibodies	Novus Biologicals NB100-134; lot AE-3
Peak calling parameters	The Homer suite (version 4.3) was used for identification of peak regions, annotation, and motif enrichment analysis. HIF1A peaks were called using the findPeaks module in factor mode with input genomic DNA controls, a local fold-change threshold of 3 (-L 3) and a false discovery rate threshold of 0.1% (-fdr 0.001). The total number of normalized sequencing tags associated with peaks common to all four lines were used to control for cell line-specific IP efficiencies (tags in common peaks) by adjusting the global fold-change over control threshold for the called peaks accordingly (-F 7 (HCT116), -F 6 (RKO), -F 13 (A549), -F 14 (H460)).
Data quality	FDR and enrichment fold change thresholds are described above.
Software	Peak enrichment signals were obtained using the Homer suite annotatePeaks.pl module (-size given). Enrichment of both known and de novo identified sequence motifs was analyzed with the findMotifsGenome.pl module. Peak to TSS distances were calculated with the annotatePeaks.pl module and the gUtils (v0.2.0) and GenomicRanges (v1.36.0) R packages. For direct comparison of peak signals across cell types, MAnorm (v1.1.4) (40) was used to normalize and quantify read densities at all peak loci (default settings). Third party ChIP-seq data (ENCODE) were downloaded raw and processed as described above.
